# Investigating Edaravone Use for Management of Amyotrophic Lateral Sclerosis (ALS): A Narrative Review

**DOI:** 10.7759/cureus.33746

**Published:** 2023-01-13

**Authors:** Purushottam Neupane, Pawan Kumar Thada, Pramod Singh, Abdul Rafae Faisal, Niraj Rai, Prabhat Poudel, Madeeha Subhan Waleed, Jonathan Quinonez, Samir Ruxmohan, Esha Jain

**Affiliations:** 1 Medicine and Surgery, Punjab Medical College, Faisalabad, PAK; 2 Research and Academic Affairs, Larkin Community Hospital, Miami, USA; 3 Neurology, Faisalabad Medical University, Faisalabad, PAK; 4 Nephrology, Faisalabad Medical University, Faisalabad, PAK; 5 Psychiatry, Punjab Medical College, Faisalabad Medical University, Faisalabad, PAK; 6 Surgery, Nepal Medical College, Kathmandu, NPL; 7 Internal Medicine, Lower Bucks Hospital, Bristol, USA; 8 Neurology/Osteopathic Neuromuscular Medicine, Larkin Community Hospital, Miami, USA; 9 Neurocritical Care, Unviersity of Texas Southwestern Medical Center, Dallas, USA; 10 Neurology, Larkin Community Hospital, Miami, USA; 11 Family Medicine, Cooper University Hospital, Camden, USA

**Keywords:** treatment choices, adult neurology, neurology and critical care, amylotrophic lateral sclerosis, pathophysiology of als

## Abstract

The use of Edaravone, given orally, for the treatment of amyotrophic lateral sclerosis (ALS) was officially approved by the Federal Drug Association (FDA) in 2017. ALS is a rare and progressive degenerative disease that worsens over time. It attacks and destroys the nerve cells that control voluntary muscles, thus leading to weakness, eventual paralysis, and, ultimately death. Edaravone was given initially intravenously, but recent evidence shows better results with oral suspension. This narrative review is aimed to investigate the benefit of Edaravone for the management of ALS, compare it to Riluzole, discuss its mechanism of action, route of use, and side effects, and ultimately discuss future implications of this pharmacotherapy.

## Introduction and background

Amyotrophic lateral sclerosis (ALS) is a progressive degenerative disease that affects motor neurons in the brain and spinal cord. It results in the deterioration and loss of motor neurons, ultimately leading to paralysis and death in the afflicted patient [1]. While there is no curative pharmacotherapy for ALS, Riluzole was the only Federal Drug and Administration (FDA) approved drug in the 1990s. Edaravone, a recently FDA-approved drug in 2017, is effective in mitigating the progression of ALS during the early stages of the disease [2]. It is administered through intravenous (IV) infusion 10 times per month [2]. The route and frequency of this drug can sometimes be problematic for patients. Compared to Riluzole, which increases a patient's survival, Edaravone works by slowing down the disease progression measured by a test that evaluates motor function [2]. Edaravone is an antioxidant that removes oxygen free radicals and nitric oxide, as oxidative stress plays a prominent role in neuronal cell death [3].

Four randomized clinical trials have studied the efficacy and safety of Edaravone with promising results. Edaravone's dosing does not need to be adjusted for hepatic or renal impairment and does not interact with other drugs; thus, it is generally safe [4]. Edavarone has been proven to delay the degeneration of motor neuron dysfunction in the brain and spinal cord, allowing for the prolonged survival of patients. Furthermore, Edavarone treatment was associated with significantly less functional decline compared to placebo [4].

With Edaravone being a recently approved medication for the treatment of ALS, it is an effective drug, especially in the early stages of disease progression. It has been proven to be quite efficacious in patients with preserved vital capacity and shorter disease duration [3-4]. It will be beneficial to conduct further studies that analyzes the efficacy of Edavarone in the later stages of ALS as information is limited.

## Review

Define/Discuss ALS

Amyotrophic lateral sclerosis (ALS) (also called Lou Gehrig's disease) is a rare progressive motor neurodegenerative disease that involves the brain and spinal cord, causing loss of voluntary muscle control. Both sporadic and familial ALS (FALS) are indistinguishable clinically. The sporadic type is more common, with 90%-95% of cases classified as sporadic. It involves both upper and lower motor neurons, progressing to muscle atrophy, paralysis, and respiratory insufficiency, inevitably, death within two to five years. Initially, there is an asymmetric weakness in the disease process, but later on, more muscles become affected until there is a symmetric distribution of weakness in all regions. With prominent corticospinal involvement, sometimes hyperactivity of the muscle-stretch reflexes predominates with muscle rigidity, often out of proportion to weakness. Rarely familial ALS develops concurrently with frontotemporal dementia. Bowel and bladder, sensory, and cognitive functions are usually preserved. Although the incidence varies between countries and regions, the average incidence globally is 1.6 per 100000 cases, and higher among Caucasians. Further, the incidence of ALS increases with age, likely due to increased life expectancy and better diagnosis [5-6]. Although a definite, single etiology is unknown, multiple potential mechanisms have been proposed. Degeneration and gliosis of motor neurons in the spinal cord and loss of Betz cells in the motor cortex have been reported. Aggregation of misfolded proteins due to mutation genes encoding SOD1 (superoxide dismutase 1), TDP-43 (TAR-DNA binding protein-43), and FUS/TLS (fused in sarcoma/translocated in liposarcoma) results in endoplasmic reticulum stress. TDP43 and FUS play multiple roles in controlling cell proliferation, transcription, DNA repair, and translation. SOD1 mutations lead to unchecked free radical toxicity, cascading overwhelmed inflammatory response resulting in neuronal injury, and eventually apoptosis [7]. FALS also involves numerous genetic mutations, most commonly C9ORF72 (open reading frame 72 on chromosome 9). Hexanucleotide repeat expansions account for around 45%-50% of FALS [8]. In a few cases, defective axonal cytoskeleton and transport is the primary problem.

Edaravone mechanism of action and use in ALS

**Figure 1:**
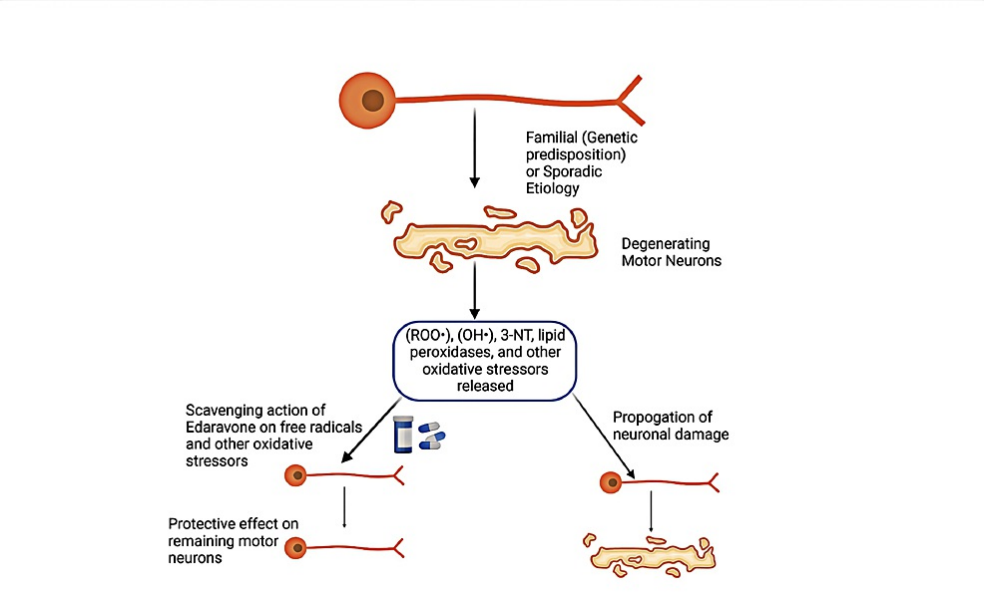
Proposed mechanism of Edaravone in the management of ALS. Image created by author PS. ALS, amyotrophic lateral sclerosis

In both familial and sporadic cases of ALS, oxidative stress is a prime contributor to the progressive degeneration of motor neurons [7-9]. This phenomenon is evidenced by increased levels of 3-nitrotyrosine (3-NT), a marker of oxidative injury, in the cerebrospinal fluid. In addition, lipid peroxides, peroxyl nitrite, and hydroxyl radicals have also been ascribed to contribute to the disease process [9]. Edaravone, a free radical scavenger, reduces hydroxyl, peroxyl, and 3NT levels [9]. The protective nature of Edaravone on glial cells, endothelial cells, and neurons against oxidative stressors has been well documented [10]. In an open-label phase II trial (MCI186-12) involving ALS patients, Edaravone, 30 mg to five subjects and 60 mg to 20 subjects, was administered in six cycles of two weeks, once daily IV dosing, with an intervening two-week gap in between each cycle. The level of 3NT in cerebrospinal fluid (CSF) started to fall after the first treatment cycle and was almost undetectable at the end of the treatment duration. This decrease in 3NT levels is significantly correlated with a much slower reduction in ALS functional rating scale ALSFRS-R score during the research compared to the six months before Edaravone treatment (p = 0.039, difference = 2.4-3.5 points, Wilcoxon signed rank test) [11]. Figure 1 depicts the presumed mechanism of Edaravone on ALS (see below).

Side effects/contraindications of Edaravone

Edaravone is contraindicated in people with a reported history of hypersensitivity reactions to the drug. If such a reaction occurs, the administration of Edaravone is ceased immediately, and the patient is managed symptomatically until the effects wear off. Similarly, sulfite-associated allergic reactions may also occur with sodium bisulfite as a constituent. Because of this, patients should be monitored for signs of hypersensitivity and anaphylaxis [12].

No studies exist on potential fetal developmental risks in pregnant mothers. Any effects on breast milk or its production are also unknown. No extra precautionary measures have yet been advised for its use in the elderly population. Finally, this drug is not contraindicated in renal or hepatic impairment patients [13-14]. The most commonly reported side effects include bruising and gait disturbances. They are followed by headaches, skin-related conditions (itching, blisters, reddening, rash, dermatitis, and eczema), and glycosuria in a few cases [14-16]. Nevertheless, based on the data that have been made available, Edaravone is generally considered safe for use. Side effects are mild and reversible.

Pros and cons of using Edaravone for ALS

Pros and Cons of Intravenous (IV) Edaravone

Edaravone IV dosing has 100% bioavailability and high protein binding capacity, so issues that generally hinder bioavailability and oral absorption do not pose a hurdle for IV Edaravone [17]. Also, it has scope for individualized treatment [17]. IV Edaravone possesses anti-inflammatory actions against activated microglial cells and neuroprotective benefits over oxidative stress [18]. The brief elimination half-life may amplify the inconvenience of using Edaravone through the IV route, as ALS is a stable and progressive disease requiring everlasting pharmacological formulation, which decreases patient compliance [19]. The Edaravone capacity of plasma protein binding is 92%, chiefly to albumin.

Consequently, it may be expected that a pathological state like malnutrition, which declines the albumin concentrations, could potentially elevate Edaravone plasma concentration aggravating its pharmacological effect [17-20]. The drug reveals mild and unrushed advancement of ALS, but it fails to ameliorate the manifestation of ALS and bears a significant threat of kidney damage [20]. The unfavorable outcome of a parenteral formulation is hypersensitivity, infection at the infusion site, and deep vein thrombosis. Sometimes, it can cause headaches, insomnia, and transient leukopenia [19-21].

Pros and Cons of Oral Edaravone

The oral formulation has the edge over the IV route as it can be used in both pre-hospital and outpatient settings and has better patient compliance [19-20]. Solubility and stability are the greatest barrier, together with the requirement for protection against oxidative degradation and an acidic environment for oral dosage [17-20]. Edaravone has not yet been clinically studied in an oral dosage form. However, Mitsubishi Tanabe Pharma Corporation (Osaka, Japan) is currently testing it in an oral dosage form for ALS as part of phase 3b, a randomized, double-blinded, multicentric study [18-20].

Pros and Cons of Both IV and Oral Edaravone

Edaravone is believed to minimize oxidative stress, acting as a free radical scavenger considering oxidative stress has long been attributed to ALS [21-24]. The downside of Edaravone use is glucosuria (the amount of blood glucose surpasses the kidney's capacity to captivate it) [21-24]. Also, the monthly expenses of using Edaravone is more than that of Riluzole [21-24]. Furthermore, the posthoc analysis proposed that Edaravone was potent in a narrow sub-group, such as patients with gentle symptoms and brief duration of ALS [21-24]. According to the manufacturer's inspection, Edaravone has an incremental cost-effectiveness ratio of $1,957,200 per quality-adjusted life year achieved, making it unprofitable compared to the standard of care [23-24]. Likewise, IV formulation of Edaravone did not have an issue for potential drug‐drug interactions (DDI); oral administration of Edaravone needs clinical evaluation for DDI [24].

Comparison of Edaravone to other ALS treatments (Riluzole)

Edaravone’s antioxidant properties help reduce mortality by decreasing disease complications, while Riluzole is a glutamatergic neurotransmitter inhibitor that slows disease progression [25-28]. The decline in forced vital capacity (FVC) is less with Edaravone than with placebo in ALS patients [26-28]. Only Edaravone and Riluzole hold FDA approval for the treatment of ALS. Edaravone is more efficacious in slowing the early disease progression than Riluzole, which has lower efficacy on disease progression and moderate survival benefit [26-28]. Common adverse effects of Edaravone include constipation, dysphagia, and contusion. When discussing the incidence of severe adverse effects (SAEs), death, and discontinuation of Edaravone, it was the same or less compared to the placebo [27]. A clinical trial conducted in Northern Italy compared changes in the ALS functional rating scale, ALSFRS-R score, FVC, and Medical Research Council (MRC) score in three months and six months duration of 31 patients who were administered with Edaravone and 50 patients who were not treated with Edaravone concluded that the benefits of Edaravone on the treatment of ALS in Caucasian ancestry is not proven [28]. Edaravone and Riluzole are the only effective FDA-approved drugs to manage ALS. Edaravone decreases the incidence of complications, while Riluzole slows disease progression with lower efficacy [25-28].

Evidence of Edaravone used in the clinical setting

Since Edaravone's approval by the Japanese and Korean governments in 2015 and the U.S. Food and Drug Administration in May 2017 as a medication for the treatment of ALS, it has been utilized in clinical settings [29-31]. Edaravone is currently offered orally and intravenously. It can either be administered as a 60 mg IV infusion over 60 min or as a 105 mg suspension delivered orally or through a feeding tube in the morning following an overnight fast [30]. Edaravone is given intravenously (IV) or orally (oral therapy) in 28-day cycles, beginning with daily dosing for 14 days during the treatment phase and ending with a 14-day drug-free break. In subsequent cycles, there would be a 14-day drug-free interval after 10 of the 14 days of the treatment were spent taking Edaravone daily. After receiving IV therapy, patients can transition to oral therapy at the same dose and frequency [31-32]. A retrospective analysis of 45 ALS patients who entered the hospital for the first time between 2013 and 2018 was carried out in a Japanese single-center study. Twenty-two patients received Edaravone treatment for an average duration of 26.6 months, while the remaining patients received no treatment with Edaravone and were kept in the control group. The Kaplan-Meier analysis showed that the median survival lengths were 49 and 25 months in the Edaravone and control groups, respectively. The survival rate was considerably greater in the Edaravone group than in the control group, suggesting a survival benefit of the medicine [33].

The ability of Edaravone to slow the progression of ALS was not shown in an Indian observational trial with a sole emphasis on its use in ALS patients. Single fiber electromyography (SF-EMG) jitter difference between the patients improved slightly but was not statistically significant, even though the primary desired endpoint could not be met [34]. Examining the medical records of 16 ALS patients who took extended Edaravone between 2015 and 2021 in a single tertiary ALS facility allowed researchers in Korea to examine the long-term effects and safety of the drug. Patients only experienced mild side effects that were well tolerated [35]. In a survey of 67 US doctors, 3,007 patients received prescriptions for Edaravone, 67% of whom were also on Riluzole, 24% of whom had previously taken Riluzole, and 9% of whom had never taken Riluzole. Over 50 instances of drug ineffectiveness were recorded. In a surveillance assessment of Edaravone initiated by the United States Department of Veterans Affairs after its approval in 2017, the analysis compared Edaravone (alone or with Riluzole) with Riluzole only. Although Edaravone reduced the death rate, the change was not statistically significant [36].

 In a 72-week-long Kuwaiti study to assess the effectiveness and safety of Edaravone in ALS patients at a tertiary neurology clinic, 17 ALS patients were evaluated, and Edaravone infusion was given as per drug protocol. After one year of Edaravone therapy, a considerable functional deterioration was seen. Despite the medicine appearing to be relatively safe and well-accepted by all patients, there was a high degree of dissatisfaction among the cohorts [37]. Individuals with more severe disease, longer disease duration, and slower disease progression may also benefit from treatment with Edaravone, according to a post hoc analysis of data from both MCI 186-19 randomized, controlled, open-label investigations [38]. Depending upon clinical conditions and the healthcare systems, there is a huge discrepancy among the results of different countries regarding the efficacy, safety, and tolerance of Edaravone.

Clinical implications and future perspectives of using Edaravone for ALS

Amyotrophic lateral sclerosis is a multifactorial neurological disorder with genetic and environmental risk factors. Despite the ongoing trials and the new advancements in medicine, ALS remains incurable. Riluzole is the primary drug used for ALS. However, the efficacy of this drug remains limited. Edaravone is the new drug approved for ALS. Non-pharmacological treatments like stem cell therapy are also well tolerated in patients with ALS. The earlier the diagnosis of ALS, the earlier the initiation of treatment, and more clinical trials recruitment for future studies to improve patient outcomes. However, adequate research is required regarding the safety and efficacy of individual therapies or their usage in combination. The cost and efficacy of treatments are of prime importance. The patients should be fully informed about both the costs and benefits of Edaravone while receiving the drug, and the goals of care should also be taken into account and discussed with the patient. Different mechanisms affecting the progression of ALS have not yet been determined. Edaravone plays a vital role in hindering the progression of motor function deterioration by preventing oxidative stress from causing neuronal death, hence improving morbidity and mortality associated with the disease. The FDA has approved the intravenous and oral suspension of the drug for ALS. The efficacy of Edaravone in mild-moderate and severe ALS should be studied further. Understanding the pharmacodynamics and pharmacokinetics of Edaravone is crucial, and the determination of this drug's safety and clinical outcomes in a real-world setting should be investigated. Furthermore, randomized clinical trials involving large general populations under placebo-controlled conditions should be done to overcome this neurological nihilism.

## Conclusions

Amyotrophic lateral sclerosis is a progressive, debilitating, and fatal disorder that affects hundreds of thousands of people annually. With the increasing incidence of this disease, further efforts have been aimed at mitigating this deadly disease process. The mainstay of treatment has been Riluzole. However, a newer FDA-approved drug, Edaravone, is effective in managing ALS, especially in the earlier stages of the disease. This narrative review not only compared Riluzole with Edaravone but also discussed its mechanism of action, route of use, and clinical implications of Edaravone for the future. More extensive research is required to assess the efficacy of Edaravone in the middle to later stages of ALS with the hopes that Edaravone can prove to be an effective treatment modality.
